# Facilitators and barriers of maternal and infant healthcare access for undocumented migrants in the first 1000 days of life: a systematic review of the literature

**DOI:** 10.1080/26410397.2025.2560189

**Published:** 2025-09-17

**Authors:** Caterina Montagnoli, Nathalie Bettina Neeser, Bernice Simone Elger, Tenzin Wangmo

**Affiliations:** a PhD student, Institute for Biomedical Ethics, University of Basel, Basel, Switzerland; Research Fellow, Midwifery Degree Program, Geneva School of Health Sciences, University of Applied Sciences and Arts of Western Switzerland, Delémont, Switzerland.; b Postdoctoral Researcher, Institute for Biomedical Ethics, University of Basel, Basel, Switzerland; c Head of the Institute, Professor, Institute for Biomedical Ethics, University of Basel, Basel, Switzerland; Professor, Unit for Health Law and Humanitarian Medicine, Center for Legal Medicine, University of Geneva, Geneva, Switzerland; d Professor, Institute for Biomedical Ethics, University of Basel, Basel, Switzerland

**Keywords:** undocumented immigrants, maternal healthcare, first 1000 days, infant healthcare, access to healthcare

## Abstract

Adequate access to maternal and infant healthcare in the first 1000 days of life is concurrent with lifelong well-being and should be guaranteed regardless of nationality, legal status, and social conditions. By exploring how migration status affects the social determinants of health, this review provides a nuanced understanding of the barriers and facilitators encountered accessing healthcare by undocumented women and their infants in the first 1000 days of life. Following the PRISMA guidelines and the registered protocol (CRD42022328220), a literature search was conducted in PubMed, Embase, CINAHL, PsycInfo, and Scopus. The search focused on manuscripts including undocumented migrants as study participants and reported direct or indirect data on the utilisation or outcomes of maternal and infant healthcare within the defined first 1000 days of life. Fifty-two studies met the inclusion criteria. A narrative synthesis was performed to summarise the information obtained from the data extraction process. Identified barriers include legal barriers (e.g. criminalisation of migrants or complexity of administrative procedures) and socio-cultural barriers (e.g. inadequate health literacy and financial challenges). Facilitators encompassed legal facilitators, like dedicated healthcare clinics, and socio-cultural facilitators, such as language support and healthier lifestyle habits. Ethical issues in connection to healthcare access of undocumented migrants in the first 1000 days were also mapped from the included papers. Considering the health consequences on future generations, the economic implications, and the vulnerability of migrant women, the findings offer positive examples that could be put into place to move towards universal health coverage through a holistic approach that shifts from exclusion and criminalisation to support and compassion. *DOI: 10.1080/26410397.2025.2560189*

## Plain language summary

Good healthcare for mothers and babies during the first 1000 days of life is essential for lifelong health and well-being. This care should be available to everyone, no matter their nationality, legal status, or social situation. We performed a systematic review of the literature, searching five research databases and selecting 52 studies, to understand how being undocumented affects women’s and infants’ access to healthcare. The review found two main types of barriers to healthcare: legal barriers, which comprise among others complicated paperwork, and the criminalisation of migrants; and social and cultural barriers, including low health knowledge, language problems, and financial hardship. We also found factors that make access easier: among the legal facilitators, these were clinics and policies designed to serve undocumented migrants, social facilitators included translation services and healthier lifestyle habits. Some studies also discussed ethical issues, such as fairness and human rights, in giving undocumented migrants access to maternity and infant healthcare. Our findings show that supporting undocumented mothers and infants benefits both families and society. It can improve long-term health, reduce costs, and promote fairness. To achieve this, countries need to shift from exclusion and punishment to support and compassion, moving closer to universal healthcare for all.

## Résumé

Un accès adéquat aux soins de santé maternelle et infantile pendant les 1000 premiers jours de la vie favorise le bien-être tout au long de la vie et doit être garanti, quels que soient la nationalité, le statut juridique et les conditions sociales. En explorant les effets du statut migratoire sur les déterminants sociaux de la santé, cette étude permet une compréhension nuancée des obstacles et des facteurs facilitant l’accès aux soins de santé pour les femmes sans papiers et leurs nourrissons au cours des 1000 premiers jours suivant la naissance. Conformément aux lignes directrices PRISMA et au protocole enregistré (CRD42022328220), une recherche documentaire a été menée dans PubMed, Embase, CINAHL, PsycInfo et Scopus. La recherche s’est concentrée sur les manuscrits incluant des migrants sans papiers en tant que participants à l’étude et a rapporté des données directes ou indirectes sur l’utilisation ou les résultats des soins de santé maternelle et infantile au cours des 1000 premiers jours de vie définis. Cinquante-deux études répondaient aux critères d’inclusion. Une synthèse narrative a été préparée pour résumer les informations obtenues à partir du processus d’extraction des données. Les obstacles identifiés comprennent les barrières juridiques (par exemple la criminalisation des migrants ou la complexité des procédures administratives) et les obstacles socioculturels (par exemple des connaissances insuffisantes en matière de santé et des difficultés financières). Les facilitateurs englobaient des facteurs juridiques, comme les centres de santé dédiés, et des facilitateurs socioculturels, tels que le soutien linguistique et des modes de vie plus sains. Des questions éthiques liées à l’accès aux soins des migrants sans papiers pendant les 1000 premiers jours ont également été recensées à partir des documents inclus. Compte tenu des conséquences sur la santé des générations futures, des implications économiques et de la vulnérabilité des migrantes, les résultats offrent des exemples positifs qui pourraient être mis en place pour se rapprocher d’une couverture sanitaire universelle grâce à une approche holistique qui passe de l’exclusion et de la criminalisation au soutien et à la compassion.

## Resumen

El acceso adecuado a los servicios de salud materna e infantil en los primeros 1000 días de vida va de la mano con el bienestar de por vida y se debe garantizar sin tener en cuenta la nacionalidad, la condición jurídica y las condiciones sociales de la persona. Al explorar cómo la condición de inmigrante afecta los determinantes sociales de la salud, esta revisión ofrece una comprensión matizada de las barreras y los facilitadores encontrados por las mujeres indocumentadas y sus bebés cuando acceden a los servicios de salud en los primeros 1000 días de vida. Siguiendo las pautas de PRISMA y el protocolo registrado (CRD42022328220), se realizó una búsqueda de la literatura en PubMed, Embase, CINAHL, PsycInfo y Scopus. La búsqueda se enfocó en manuscritos que incluyen a migrantes indocumentadas como participantes de estudios, e informó datos directos o indirectos sobre el uso o los resultados de los servicios de salud materna e infantil en los primeros 1000 días de vida. Cincuenta y dos estudios reunieron los criterios de inclusión. Se realizó una síntesis narrativa para resumir la información obtenida del proceso de extracción de datos. Las barreras identificadas eran barreras jurídicas (ej., penalización de migrantes o complejidad de los procedimientos administrativos), y barreras socioculturales, (ej., alfabetización inadecuada sobre la salud y retos financieros). Los facilitadores consistían en facilitadores jurídicos, como centros de salud dedicados, y facilitadores socioculturales, como asistencia lingüística y hábitos de vida más sanos. Los asuntos éticos en conexión con el acceso a los servicios de salud por migrantes indocumentadas en los primeros 1000 días también fueron mapeados de los artículos incluidos. Considerando las consecuencias para la salud en futuras generaciones, las implicaciones económicas y la vulnerabilidad de las mujeres migrantes, los hallazgos ofrecen ejemplos positivos que podrían implementarse para avanzar hacia la cobertura universal de salud mediante un enfoque holístico que reemplace la exclusión y penalización por apoyo y compasión.

## Introduction

The provision of equitable and comprehensive healthcare services is considered a fundamental human right by international organisations such as the World Health Organization (WHO), the United Nations (UN), and the World Medical Association (WMA). This commitment to ensuring universal access to healthcare transcends boundaries of nationality, legal status, and social circumstances, emphasising the principle of providing the highest attainable standard of health to every individual.^1–3^ In maternal and infant health, this commitment stands out as it concerns the well-being of both the present and future generations. In particular, it translates into care provided to mothers and their infants during the critical period spanning from conception to the infant's second year of life, known as the first 1000 days of life, which hold profound implications for the infant’s lifelong well-being.^4^ An increasing body of research demonstrates that the timeframe from conception to a child’s second year should be considered as a continuum, and that this period is acknowledged as the most important time to build strong and durable health in the long term for both mothers and infants.^5–8^ In fact, maternal and neonatal adverse health-related events, like preterm birth, have proved to influence the child’s short- and long-term developments.^9^ Studying this window of opportunity not only safeguards the health and development of the child but also yields long-term societal benefits by reducing health disparities, improving educational outcomes, and lowering the burden of disease across the lifespan.

To prevent adverse long-term outcomes, according to the WHO, women should attend at least eight antenatal care (ANC) contacts during pregnancy. For post-partum care, the WHO suggests four appointments in the six weeks following the birth for both mothers and their babies with healthcare professionals like midwives.^10,11^ Moreover, recent joint guidelines from the WHO and United Nations Children's Fund (UNICEF) stated that infants should undergo at least five well-child visits in the first year and three more during the second year of life, completing the first 1000 days of life timeframe.^12^ Of these 20 contacts in total, the literature reports that many visits are unfortunately missed particularly among disadvantaged groups.^13^ Barriers like limited finances or low health literacy, especially for migrants, make access to healthcare in the first 1000 days of life essential to ensure both immediate and long-term health for mothers and their infants.^14,15^

Individuals have migrated from one place to another, and continue to do so due to work, conflicts, natural disasters, and many other reasons. However, the nature of this movement has changed significantly over time and in light of varying national restrictions.^16,17^ Undocumented or irregular migration, or the movement of people occurring outside established regulatory norms,^18^ presents a unique challenge to achieving universal health coverage and health equity.^19^ Although undocumented migrants contribute to the wealth of the host society, they remain largely in the background and benefit rarely from services such as national health care, due to the fear of being denounced and forced to return to their country of origin.^20,21^ On the same note, research suggests that only for immediate urgent matters do undocumented migrants seek healthcare.^22,23^ Maternal and infant healthcare are among the limited situations in which undocumented migrants seek medical attention, yet their need for healthcare is frequently unmet due to the many challenges connected to their lack of legal status.^24^ According to the recent literature, undocumented migration has in fact been associated with increased occurrence of adverse pregnancy outcomes including maternal depressive symptoms as well as gestational diabetes and pre-eclampsia.^25^

Different studies have pointed to disparities in healthcare access and outcomes for undocumented migrant women in the first 1000 days of life.^26,27^ These disparities encompass multiple dimensions, ranging from reduced utilisation of contraceptive methods, leading to higher rates of unintended pregnancies, delays in seeking perinatal care, and a heightened prevalence of pregnancy and delivery-related complications.^28,29^ Moreover, undocumented migrant women frequently face social and legal obstacles that compound their challenges in accessing essential healthcare services.^30,31^ Ensuring universal access to healthcare, coverage, services, and financial protection, is one of the most fundamental yet complex challenges faced by healthcare systems, and yet no clear definition of access to healthcare is provided globally.^32^

Conversely, literature on the “healthy migrant effect”, meaning when the migrant population has “a better health status compared with others in the home country, but also in comparison with the population in the host country”,^33^ challenges what was thought to be a solid connection between poor access and poor outcomes.^34,35^ In the field of maternal and infant health, this phenomenon is known as the “Latina paradox”,^36^ after its first analysis among Latin American undocumented migrants accessing healthcare in the state of New York,^37^ where maternal and infant health outcomes of undocumented populations often surpass those of the native population.^37^ These protective effects might also wane after several years.^37^ Therefore, in maternal and infant health, it is particularly relevant to ensure healthcare access of this population to set the basis for solid long-term health for both mothers and infants. The literature is yet to provide a clear understanding of the strategies that undocumented migrant women use to overcome the barriers they are faced with to meet healthcare needs. This knowledge shortfall could be because of practical challenges associated with identifying the population of undocumented migrant women.^38^ Legal definitions of who constitutes an undocumented migrant differ from one receiving country to another. There are also many social factors and a general lack of trust in the system that make it difficult for this population to be reached and engaged in studies.^39^ The social determinants of health, which are defined as “the non-medical factors that influence health outcomes”,^40^ play a crucial role in this context. These include legal status, economic stability, and access to healthcare, all of which significantly impact the health and well-being of undocumented migrant women and their infants. There are several studies that capture maternal and infant health outcomes,^23,41,42^ but the knowledge provided in the literature around facilitators and barriers to accessing healthcare in the first 1000 days remains scattered.

For this reason, we conceptualised the following research question “What are the facilitators and barriers for undocumented migrant populations in accessing maternal and infant healthcare during the first 1000 days of life?”. In response to this question, our review aimed to bring together the existing knowledge concerning barriers and facilitators of access to maternal and infant healthcare in the specific timeframe of the first 1000 days of life. By examining how migration status affects the determinants of health and thus healthcare access and health outcomes, this review seeks to provide a nuanced understanding of the challenges faced by this vulnerable population, while also identifying strategies and interventions that can be implemented via specific policies and improve access to equitable healthcare services for underserved populations.

## Methods

In this section, we present the steps undertaken to address the research question in accordance with the Preferred Reporting Items for Systematic Reviews and Meta-Analyses (PRISMA) guidelines.^43,44^ The protocol of this systematic review has been registered in PROSPERO and can be accessed through its registration number CRD42022328220. After a number of preliminary searches to evaluate the key terms, a systematic literature search was conducted on PubMed, Embase (no Medline), CINAHL, PsycInfo, and Scopus. Despite the growing amount of grey literature in systematic reviews in the health sciences and in health services research, we decided to focus only on published peer-reviewed articles. The final searches in each database were performed on 20th February 2023 and updated on 1st November 2024. In Table 1, we present the search terms used according to an adaptation of the PICO (Population, Intervention, Context and Outcome) framework to address our specific search interest.

**Table 1.** Search strategy and terms

		TERMS
**P**	Undocumented mothers and their children	undocumented immigrant; unregistered person; unregistered migrant; illegal immigrant; undocumented migrant; undocumented immigrant; unregistered immigrant; illegal migrant; irregular immigrant; irregular migrant; unauthorised immigrant; unauthorised migrant; unauthorised person; unauthorized immigrant; unauthorized migrant; unauthorized person; undocumented wom*; illegal wom*; irregular wom*; undocumented child; sans papier*; clandestin*
**C**	In an irregular legal situation	lack of legal status; paperless
**O**	Maternal and infant health outcomes in the first 1000 days timeframe	maternal health; maternal health services; maternal-child health centres; child health; infant care; child health services; maternal-child health services; prenatal care; postnatal care; perinatal care; postpartum period; pregnancy; reproductive health; reproductive health services; obstetrics; maternal care; maternal healthcare; prenatal healthcare; antenatal care; antenatal healthcare; postpartum care; postpartum healthcare; postnatal healthcare; perinatal healthcare; perinatal health; child healthcare; child health care; infant healthcare; pregnancy care; pregnancy healthcare; maternity service; reproductive health; reproductive health service; maternal health service; puerperium; pregnancy health; maternity care

The initial database search was performed in PubMed, taking notice of truncation symbols, and connecting the search terms with the Boolean operators OR and AND. After close considerations following a series of initial searches, we decided not to connect different concepts with the adjacent (“adj”) operator because – after checking – it added no further specific literature to the search. The search was then extended to the other four databases, with database-specific adjustments whenever necessary. MeSH terms were exploded (“exp”) and all subheadings were included to ensure that the selected search terms as well as more narrow terms were retrieved. The detailed search query can be found in the Supplementary Material file. To assure the quality of the search results, completeness and transparency, an additional Google Scholar search was performed using the combination of the research terms presented in Table 1.

Studies were deemed eligible for inclusion if they (i) were published between 1st January 2000 and 31st October 2024; (ii) were published in English, German, Spanish, Italian, or French; (iii) the setting was one or more of the OECD countries; (iv) reported primary, peer-reviewed, original qualitative or quantitative data; (v) focused or specifically included undocumented migrants as study participants; and (vi) reported direct or indirect data on the utilisation or outcomes of maternal and infant healthcare within the defined first 1000 days of life. We chose to focus exclusively on recent (2000–2024) literature from OECD countries to enable meaningful comparisons across healthcare systems in medium- to high-income settings with recent developments of undocumented migration dynamics and global health policies. In fact, OECD nations are characterised by relatively stable governance and healthcare systems that have some capacity to integrate undocumented migrants. While undocumented migrants are also present in non-OECD settings, these are often shaped by fundamentally different conditions (e.g. camps managed by UNHCR or NGOs such as MSF), making direct comparison inappropriate for the scope of this review.

Studies were excluded if they (i) were published outside the identified time period; (ii) did not report primary, peer-reviewed data (such as scoping or systematic reviews, editorials, committee opinions); (iii) did not include undocumented migrants, in particular pregnant women and their infants as their study participants; (iv) were outside the first 1000 days’ time frame; (v) were normative studies; (vi) were not available in full text; or (vii) did not concern human health. Articles focusing on access to sexual health were also excluded whenever maternal care or infant care were not concerned.

Title and abstract screening as well as full-text screening and data extraction were all performed on Covidence,^45^ an online software for managing and streamlining systematic reviews. Following the searches, all articles were uploaded on Covidence, where an automatic identification of duplicates was carried out. On the basis of the pre-defined inclusion and exclusion criteria, two reviewers (CM, NN) independently performed the titles and abstracts screening. In case of disagreement in the selection process, a third reviewer (TW) was consulted. Studies were only included if consensus was reached by all authors.

We then performed a full-text screening to assess the articles retrieved following a data extraction protocol. Two reviewers (CM, NN) extracted these data, which were then downloaded from Covidence into an Excel file. Key data collected included general characteristics of the studies, the overall methodological approach and study design, the sample size, time indication, the study’s objective, information on the studied population (mothers, infants or both), the study results related to the use and outcomes of maternal and infant healthcare services by undocumented migrants as well as the cultural, ethical, social, and legal facilitators and barriers the undocumented migrants were confronted with. A third author (BE) performed a data checking on a random ∼20% of all extracted articles (*N* = 10).

A qualitative and a quantitative results’ narrative synthesis was then performed to summarise the information obtained from the data extraction process. Inspired by Popay et al directives for narrative synthesis in systematic reviews,^46^ the initial data extraction was followed by a preliminary synthesis “exploring relationships in the data and assessing the robustness of the synthesis” (p.11). We then categorised the synthesised data into barriers and facilitators.

To assess the quality of quantitative and qualitative studies, we used specific CASP quality assessment tools^47^ for these two study types. We assigned a quality assessment score to each article that underwent data extraction. Articles whose quality assessment score was inferior to 65% (i.e. less than 7/11 for qualitative and less than 8/12 for quantitative studies) were excluded. The quality of the systematic review itself was ensured as PRISMA reporting guidelines were followed.^43^

### Positionality

This systematic review was conducted by four highly educated cis-women. Professionally speaking, all authors have different backgrounds, including midwifery, medicine, gerontology, anthropology, gender studies, and theology; and currently work in the context of biomedical ethics. The ethnicities and nationalities of authors vary, with several of them residing outside of their countries of origin. Additionally, three out of the four authors are mothers, with their children varying greatly in age.

We acknowledge that our personal and professional backgrounds may have influenced our perspectives. To minimise potential biases, we followed the PRISMA guidelines for conducting and reporting systematic reviews and used CASP quality assessment tools to critically appraise the quality of included studies. Additionally, we engaged in multiple reflexive discussions, ensuring that conclusions were grounded in the extracted data and supported by the existing literature. All authors contributed to the interpretation of findings and the discussion of their implications.

## Results

**Figure 1.**
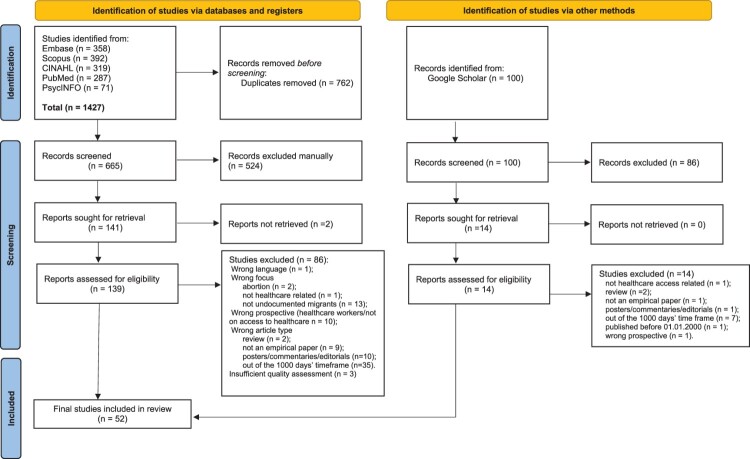
PRISMA flow diagram

In Figure 1, we present a flow chart to summarise the article selection process. The search resulted in a total of 1295 studies from Embase (*n* = 358), Scopus (*n* = 392), CINAHL (*n* = 319), PubMed (*n* = 287), and PsycInfo (*n* = 71). After removing duplicates, 665 studies were eligible for further abstract and title screening. During this process, we excluded 524 studies, resulting in 139 entries whose full texts were retrieved (full texts were not found for two studies). The 139 full texts were assessed again for eligibility against the inclusion and exclusion criteria and appraised for study quality. We excluded a total of 86 studies for the following reasons: wrong language (*n* = 1); wrong focus (abortion, *n* = 2; not healthcare related, *n* = 1; not focusing on undocumented migrants, *n* = 13); wrong perspective (healthcare workers/not on access to healthcare, *n* = 10); wrong article type (review *n* = 2; not an empirical paper, *n* = 9; posters/commentaries/editorials, *n* = 10); out of the 1000 days’ time frame (*n* = 35); insufficient quality assessment (*n* = 3). A total of 52 articles were left for data extraction. No additional study was included following the screening of 14 studies resulting from screening the first 100 articles retrieved for assessment from a Google scholar search. Fifty-two articles were included in the final analysis.

### Study characteristics

Our analysis of the 52 included studies from OECD countries reveals that the majority (*n* = 37) employ quantitative research methodologies, while 14 are qualitative studies and one study utilises a mixed methods approach (Table 2). The sample sizes across these studies exhibit a wide range, from a single participant in qualitative ethnographic research^41^ to over 3.9 million participants in quantitative research.^48^ The interest in this population appears to have evolved over time. Prior to 2018, the annual publication count remains relatively low with a maximum of three publications per year. From 2018 onwards, there are between four and seven publications annually. In the last column of Table 2, we report the country/region of origin or the ethnicity of the population as reported in the included studies. The literature employs various terms to refer to this population. In this table, we document the terminology utilised by each author in their respective articles. However, to ensure consistency in this manuscript, we choose to mainly use the wording “undocumented migrant(s)” to refer to the population of interest. The majority of included studies focus on maternal access to healthcare, with 42 out of the 52 publications putting a strong emphasis on antenatal care. While maternal and fetal as well as neonatal and early infancy health are closely related, only three studies^49–51^ have extended their focus beyond the pregnancy period and the moment of childbirth, also addressing healthcare access after the birth of the child.

**Table 2.** Description of included studies (*N* = 45)

First author’s name and year of publication	Host Country	Methodological approach and design	Sample size or available participant characteristics	Undocumented migrant’s country or region of origin (or ethnicity if country not named)
Amuedo-Dorantes 2022	United States	Quantitative, retrospective cohort study	1,201,287 births	Mexico or Central America
Atkins 2017	United States	Quantitative, retrospective, longitudinal cohort study	6262 undocumented women	Hispanic ethnicity (73.3%)
Atkins 2018	United States	Quantitative, observational study	20,876 unauthorised women	Hispanic ethnicity (27.4%)
Barkensjö 2018	Sweden	Qualitative, explorative study	13 undocumented immigrant women	Macedonia, Romania, Bosnia, Albania, Somalia, Afghanistan, Serbia, Chechnya, Morocco, and Kosovo
Cantarutti 2024	Italy	Quantitative, population-based cohort study	349,753 births	Africa (5.90%); Americas (29.56%), Eastern Mediterranean (13.94%), Europe (36.26%), South-East Asia (2.35%), Western Pacific (3.55%), Stateless 8.45%
Castañeda 2008	Germany	Qualitative, participant observation and semi-structured interviews study	20 undocumented migrant men and women, 22 NGO staff, 10 physicians, and nine local experts	Southern and Eastern Europe, Southeast Asia (esp. Vietnam), West Africa, and the former Soviet Republic
Castañeda 2009	Germany	Qualitative, narrative analysis of case studies	183 migrant clinic attendees	55 countries grouped into seven categories: post-socialist nations that have joined the EU since 2004, post-socialist, non-EU nations, Asia, Middle East, Sub-Saharan Africa, Latin America, and North America
Dos Santos 2015	Costa Rica	Qualitative, In-depth interview and participant observation study	10 healthcare professionals	Nicaragua
Eick 2022	Norway	Quantitative, retrospective cohort study	500 undocumented women	Sub-Saharan Africa (37.6%), the European Economic Area (24.9%), and the East Asia & Pacific region, (16.3%). The women originated from 73 different countries. Most women originated from Romania, Somalia and Mongolia
Eick 2023	Norway	Quantitative, cross-sectional study	829 consultations	Europe & Central Asia (10.7%), Middle East & North Africa (15.1%), Sub-Saharan Africa (46.7%), North America (0%), Latin America & Caribbean (0%), East Asia & Pacific (7.6%), South Asia (4.4%), Missing information (12.0%)
Eick 2024	Norway	Quantitative, register-based population study	5856 undocumented migrants	Missing information
Eslier 2020	France	Quantitative, prospective cohort study	9599 documented and undocumented women	North Africa (22%); Sub-Saharan Africa (16.3%) and Asia Middle East (6.5%)
Eslier 2022	France	Quantitative, prospective cohort study	9599 documented and undocumented women	North Africa (22%); Sub-Saharan Africa (16.3%) and Asia Middle East (6.5%)
Farfán-Santos 2019	United States	Qualitative, interview study	One undocumented woman	Mexico
Faurholdt 2023	Denmark	Quantitative, retrospective case-control study	882 undocumented migrants (children)	Africa (12.70%), Asia (37.87%), Europe (20.98%), North and Latin America (3.17%), Caribbean and Oceania (0.23%), Unknown (25.06%)
Fedeli 2010	Italy	Quantitative, population-based observational study	144,698 both regular and irregular migrant women	Eastern Europe (56%), Africa (20%), Asia (14%), and South America (5%)
Feldman 2021	United Kingdom	Qualitative, In-depth, structured interviews study	16 undocumented women	West Africa, North Africa, Latin America, South-East Asia and the Caribbean
Flores 2012	United States	Quantitative, cross-sectional study	196,617 migrant mothers	Mexico (81%)
Fuentes-Afflick 2006	United States	Quantitative, prospective study	3242 migrant women	Hispanic ethnicity
Funge 2020	Denmark	Qualitative, semi-structured interview study	21 undocumented women	The Philippines, Sudan, Morocco, Pakistan, Kenya, Tanzania, Uganda, and Bosnia
Funge 2022	Denmark	Quantitative, retrospective study	679 undocumented women	78 countries including the Philippines (17.8%), Uganda (5.8%), Nigeria (5.3%), Romania (5.3%) and Morocco (5.2%)
Goldade 2011	Costa Rica	Qualitative, semi-structured interview study	43 migrant women (2/3 undocumented)	Nicaragua
Held 2018	United States	Quantitative, population study	4188 undocumented women	Mexico and Guatemala
Held 2018	United States	Quantitative, retrospective cohort study	4188 undocumented women	Mexico and Guatemala
Kelaher 2002	United States	Quantitative, observational study	4173 documented and undocumented women	Guatemala, Honduras, El Salvador, Colombia, Ecuador, Mexico, Dominican Republic among others
Korinek 2011	United States	Quantitative, retrospective cohort study	387,864 births	Hispanic but origins not specified
Lane 2019	United States	Qualitative, semi-structured interviews study	56 undocumented and documented women	Mexico, Venezuela, the Dominican Republic, Guatemala, Honduras, and Colombia
Liu 2019	Sweden	Quantitative, cohort study	286,870 migrant women	31,897 from Syria, Iraq, Somali, Eritrea and Afghanistan
Loue 2005	United States	Qualitative, interview-based study	157 women	Mexico
Lu 2000	United States	Quantitative, retrospective cohort study	970 undocumented mother-child pairs	Mexico (93%)
Mamuk 2021	Turkey	Qualitative, interview-based study	236 undocumented migrant women	Middle East, Africa, Former Soviet Central Asian republic
Moinester 2024	United States	Quantitative, population-based study	4,047,067 singleton births	Mexican- and Central American-Born
Montagnoli 2023	Switzerland	Quantitative, cross-sectional study	850,288 live births	Extra-EU (9.33%), Africa (12.65%), Latin America (31.36%), Asia (17.64%), Unknown (28.97%)
Nellums 2021	United Kingdom	Qualitative, semi-structured interviews	20 undocumented women	Mainly from Africa or Asia
Reed 2005	United States	Quantitative, retrospective descriptive study	5961 undocumented mother-child pairs	Mexico (93%)
Ro 2020	United States	Quantitative, population observational study	3,991,823 live births	Non-Hispanic White, Latina, Latina Foreign-Born
Sami 2019	Switzerland	Qualitative, focus group study	33 women out of which 9 were undocumented migrants	Bangladesh, Peru, USA, Germany, Portugal, Eritrea, Brazil, Dominican Republic, Bolivia
Schoenborn 2021	Belgium	Quantitative, population-based study	871,283 unregistered mother-child pairs	South America (16%), and Eastern Europe (9.4%)
Schoevers 2009	Netherlands	Quantitative, descriptive study	100 undocumented women	Mainly from Eastern Europe and sub-Saharan Africa
Schoevers 2010	Netherlands	Mixed methods, questionnaire and semi-structured interviews	100 undocumented immigrant women	Mainly from Eastern Europe and sub-Saharan Africa
Spetz 2000	United States	Quantitative, population study	1,705,422 documented and undocumented women	Among the foreign born, the author identified Hispanic, Black, Asian/Pacific Islander, Filipinas, Mexican, Vietnamese
Sudhinaraset 2023	United States	Qualitative, in-depth, one-on-one semi-structured interviews	18 women	Taiwan, China or Mexico
Swartz 2017	United States	Quantitative, difference-in-difference study	210,200 undocumented mother and child pairs	Women in Emergency Medicaid and Emergency Medicaid Plus were predominantly Hispanic ethnicity (80% and 77%, respectively), whereas the Medicaid population was predominantly non-Hispanic (80%)
Swartz 2018	United States	Quantitative, difference-in-difference study	213,746 documented and undocumented women	Predominantly Hispanic and non-white
Tasa 2021	Finland	Quantitative, retrospective register-based study	60 undocumented women	25 different countries – the majority of participants originating from Eastern Europe and Russia (36%), Sub-Saharan Africa (20%), Asia (18%), and the Middle East (15%)
Torche 2018	United States	Quantitative, difference-in-difference study	5,532,146 women	Latin America
Vanneste 2020	Belgium	Quantitative, retrospective observational study	1619 migrant women	Sub-Saharan Africa (36%), Maghreb or Egypt (24%), Eastern Europe (29%), Sub-Saharan Africa (26%)
Vega-Gutierrez 2020	Colombia	Quantitative, longitudinal and descriptive study	563 undocumented mother-child pairs	Venezuela
Wendland 2016	Denmark	Quantitative, prevalence study	219 undocumented women	Eastern-Europe countries and sub-Saharan Africa
Wilson 2022	United States	Quantitative, historical cohort study	693 women	Hispanic (74%)
Wolff 2005	Switzerland	Quantitative, demographic study	134 undocumented women	Majority of Latin American origin
Wolff 2008	Switzerland	Quantitative, prospective cohort study	394 out of which 161 were undocumented women	Majority of Latin American origin

### Barriers preventing healthcare access for undocumented mothers and their children

In Table 3, we present the barriers to undocumented migrant women accessing healthcare in the first 1000 days. Barriers identified (*N* = 11) are divided into several categories within legal and socio-cultural barriers. This categorisation of barriers into “legal” and “socio-cultural” draws on the thematic synthesis approach described by Popay et al, which emphasises identifying patterns and relationships within the data. Through this lens, barriers were grouped based on their nature and how they were described across the studies: legal barriers often related to documentation status, criminalisation, and formal entitlements, whereas socio-cultural barriers encompassed issues such as stigma, fear stemming from legal insecurity, and lack of social support or protection.

**Table 3.** Barriers of access to maternal and infant’s healthcare for undocumented migrants

	Thematic barriers	Ref.	Example or general description
Legal	**Criminalisation of undocumented migrants**	(1–7)	Exposure to restrictive laws intended to make life difficult for unauthorised migrants (e.g. Arizona’s Senate Bill 1070) to activate a physiological and behavioural stress response (Torche 2018)
	**Complex administrative procedures and lack of social protection**	(1,8–20)	Migrant women described difficulties understanding the administrative procedures of the Swiss health care system. These difficulties were in some situations closely related to a lack of health literacy on the patient side or a lack of information (Sami 2019)
			Exclusion or restrictions on migrants' eligibility of pregnant undocumented migrants from integration policies/social protection
	**Poor working and living conditions**	(13,16,21–23)	Exclusion of undocumented migrants from employment, social welfare, and adequate housing force them into poor living conditions
Socio-cultural	**Socio-demographic factors**		
	*Education and health literacy*	(1,6,7,9,10,12,17–19,23–35)	Sociodemographic factors, clinical characteristics, lifestyle habits, dialects, and literacy levels, cultural barriers, low education, ethnicity, culture, socioeconomic status, cultural differences affecting access to care. Inadequate information and lack of information, unfamiliarity with the healthcare system
			Many did not return to learn the results of tests including blood type, Rh factor, hepatitis B and HIV status, and screening for anaemia and gestational diabetes as they were not equipped with the right means to interpret the potential results (Castañeda 2009)
			Confusion among clinic staff and healthcare providers regarding undocumented patients handling (Spetz 2000)
	*Age*	(55,60)	Advanced maternal age and adverse outcomes (Schoevers et al., 2009)
	*Language*	(55,60)	Language barriers, feelings of mistrust, and distrust towards interpreters (Barkensjö 2018)
	**Network extension**	(6,14,36,41)	Limited network extent due to recent migration
	**Economic arriers to access healthcare**	(1,3,8,10–14,16,22,24,28,33,35,39,40,42–45)	Including: adverse socioeconomic conditions, the need to pay for services out-of-pocket, and cost of healthcare
	**Fear and discrimination**	(1,3,8,11–13,15,16,18,19,33,35,37,38,46–50)	Uncertainty about the future, separation from family or social isolation, social stigma and discrimination in healthcare for undocumented migrants
			Experiences of neglectful clinical encounters, influence of healthcare professional treatment on appointment attendances (Barkensjö 2018)
			Fear and vulnerability in accessing reproductive health care due to immigration enforcement and risk from localized immigrant policing. Fear of deportation affecting healthcare decisions in the context of immigration enforcement (Lane 2019)
			Alienating discourses and policies that prioritize individuals over families and communities, work together to hinder undocumented migrants and their families from accessing essential healthcare services and the health security they need to prevent serious health conditions (Farfán-Santos 2019)
	**Limited/Lack of primary healthcare access(39)**	(1,3,17,17,31,41,42,47)	Limited availability of free healthcare services for undocumented migrants (Funge 2020)
	**Psychological factors and ambient stressors**	(1,9,10,15,16,22,40,51)	Restricted agency, intersecting stressors, and a cycle of ongoing precarity in accessing healthcare (Nellums 2021)

Legal barriers, such as the criminalisation of undocumented migrants, often prevent women from accessing timely and adequate care in the first 1000 days of life, making teenage pregnancies and complications during pregnancy more frequent.^52^ In fact, when criminalisation of undocumented migrants is cited as a barrier by a given study, authors state that access to all forms of primary healthcare like family planning services is restricted, which often results in ante-partum hospitalisation, miscarriages, and induced abortion^53^ as well as delays in accessing antenatal care^27^ and, as mentioned, teenage pregnancies. Infants whose mothers were exposed to more intense anti-immigration law enforcement during pregnancy are reported to have worse birth outcomes, including low birth weight and preterm birth.^54,55^

Similarly, complex administrative procedures to obtain affordable care prevent access to healthcare and are reported together with delayed and irregular antenatal care,^41,56^ higher risk of severe maternal morbidity^57^ and a higher proportion of hypothyroidism.^58^ Infants of mothers whose access is prevented via complex administrative procedures report higher rates of low birth weight^56,58^ and preterm birth.^58^

As per the socio-cultural barriers (*N* = 9), we have found three major categories of obstacles to access healthcare in the first 1000 days of life. Problems connected with financing access to healthcare are present in 15 studies. Cultural or educational components are also major socio-cultural barriers to healthcare access with additional maternal and infant outcomes ranging from complications during pregnancy^27^ to poor self-rated health before pregnancy.^52^ Additional maternal outcomes are unintended pregnancies,^35^ obesity,^42^ stress, depression, and extreme anxiety during pregnancies.^31^

Inadequate access to antenatal care is present whenever fear connected with the legal situation, lack of social protection and major socio-cultural barriers are cited.^27,35,42,52,56,59–63^ The consequences of inadequate antenatal care are also often connected with complications during pregnancy^27^ and lower prenatal care screening for sexually transmitted infections (STIs).^14^ Preterm birth,^35,58,64,65^ low birth weight,^56,58,64,65^ worse fetal outcomes and perinatal mortality^65^ are also outcomes cited together with this legal barrier.

Finally, it is worth noting that issues around health literacy, or the knowledge around health and the host country’s healthcare system, is cited in 15 out of the 52 studies together with the following additional adverse maternal and infant outcomes: higher rates of anaemia, inadequate weight gain, higher rates of labour complications^27^ including peripartum haemorrhage.^49^ In five included articles, no barriers are discussed.^29,30,51,66,67^

#### Ethical considerations in the first 1000 days of life

In Table 4, we present the ethical considerations related to maternal and infant healthcare access for undocumented migrants in the first 1000 days of life. The transposable nature of bioethics requires specific reflections that we report in the following paragraph.

**Table 4.** Ethical considerations in relation to maternal and infant healthcare access for undocumented migrants in the first 1000 days of life

Bioethical principles	Thematic ethical considerations	Ref.	Examples
**Justice and non-maleficence**	Consequences for future generations	^7,20,23,27^	Disadvantage of new-borns in terms of health status of undocumented mothers lacking access to free antenatal care (Atkins 2018)
**Autonomy**	Ethical practices	^2,10,25,35,40,52^	Obligation of professional confidentiality in the Netherlands, which prohibits doctors from reporting undocumented migrants to immigration authorities (Schoevers 2010)
			Ethical questions connected to research conducted on vulnerable population such as women with insecure or no legal immigration status (Feldman 2021)
**Autonomy and justice**	Barriers to healthcare access	^15,41,42,47,49,53, 56,57,60,62,63,65,70^	Some healthcare professionals refuse to accept patients covered by the state medical assistance system (Eslier 2020)
			Authors highlight the potential long-term consequences of institutional barriers during pregnancy on infants’ health and development (Torche 2019)
			Providing free and systematic access to pregnancy screening aligns with international treaties ratified by the Danish government, ensuring equal and equitable access to healthcare for undocumented migrants, including both prenatal and postnatal care, as well as the right to health for children of undocumented migrant mothers (Wendland 2016)
			Authors stress the importance of access to preventive and timely healthcare for public health (Tasa 2021)
			Authors discuss the ethical implications of upfront charging for healthcare and its impact on undocumented migrant women in England (Nellums 2021)
**Non-maleficence and beneficence**	Implicit bias and discrimination	^13,14,26,37,43,50^	National authorities do not always implement the actions required to maintain the right to access health care across Europe (Vanneste 2020)
			Experiences of withheld emergency care and discrimination faced by undocumented migrants (Barkensjö 2018)
**Justice, autonomy and non-maleficence**	Vulnerability of (undocumented) pregnant women	^8,12,16,22,39^	Dignified conditions during pregnancy, regardless of migratory status (Vega-Gutierrez 2020)
			Promotion of tubal ligations among Nicaraguan migrants in Costa Rica and its implications (Goldade 2011)

Ethical considerations are discussed with varying depth of exploration in the included papers. Twenty one out of the 52 included studies do not discuss ethical dimensions associated with healthcare access of undocumented migrants. We report the identified ethical considerations starting from the four principles of biomedical ethics^68^ – meaning justice, non-maleficence, beneficence, and autonomy – and then divided them into five categories. Justice and non-maleficence are the principles most often discussed. Relevant examples of such ethical concerns include but are not limited to (a) the health consequences for future generations that suffer from disadvantages even before their birth^69^; (b) the economic and long-term consequences of barriers to healthcare access on both societies and individuals^14,59^; (c) the implicit bias and discrimination that prevent equitable access to care and feelings of undeservingness or distrust^50,70^; and (d) the vulnerability of pregnant migrant women as subjects of unjust policies that promote sterilisation among vulnerable populations as a means to control them.^26^

### Facilitators to promoting healthcare access for undocumented mothers and their children

In Table 5, we present the facilitators of access to healthcare in the first 1000 days of life. We have divided the facilitators into two sub-categories identifying overall eight major themes with two legal and six socio-cultural facilitators of healthcare access.

**Table 5.** Facilitators of access to maternal and infant’s healthcare for undocumented migrants

	Thematic facilitator	Ref.	Examples
Legal	**Healthcare costs coverage**	(6,13–15,18,19,23–25,27,29,35,36,38,48,54)	Healthcare providers receive reimbursement for unmet costs for undocumented migrants (Schoevers 2010).
			Healthcare insurance expansion covers prenatal care, reducing complications in pregnant women and poor birth outcomes (Atkins 2018).
			Public policies aimed at providing low-/no-cost prenatal care (Atkins 2017).
	**Enforcement of health rights**	(2,3,5,8,12,16,17,21,22,28,37,53)	Policy changes that gave undocumented women formal rights to antenatal care (Eick 2022).
			Access to driver licenses or driver privilege cards in Utah for undocumented migrants allows them to reach dedicated clinics abroad (Korinek 2011).
			Pregnant women, regardless of migratory status, are considered a prioritized vulnerable group in Colombia (Vega-Gutierrez 2020).
			Acute care is assured for undocumented migrants during pregnancy and the first 6 months after childbirth, and they cannot be deported from the country during this period (Fedeli 2010).
	**Employment**	(15)	Pro-employment aspects of the Personal Responsibility and Work Opportunity Reconciliation Act (PRWORA) (Fuentes-Afflick 2006).
Socio-cultural	**Dedicated clinics**	(9,17,22,26,34,36,38,39,42,43,49,53)	National Red Cross provides primary health care, including maternity care, to undocumented migrants through a healthcare clinic. Authors state that the women felt safe in this clinic and knew they would not be reported to authorities (Funge 2020).
			Facilities where protection is guaranteed (Wolff 2008).
			NGO clinics serve as entry gates into public care, and facilitate familiarity with other healthcare systems (Eick 2022).
			Availability of health care services, the possibility of calling doctors, and access to medical care for undocumented migrants (Sami 2019).
			Fear reduction through mobile medicine and support from community-based health care (Lane 2019).
	**Socio-demographic factors**		
	*Language*	(6,13,28,33,40)	Professional interpretation and support from different backgrounds (Tasa 2021).
	*Age*	(5,14,16)	Younger age and less adverse outcomes (Vanneste 2020).
	*Education and health literacy*	(5,22,28,34)	High educational background (Wolff 2005).
			Healthier patterns in alcohol, tobacco, and substance abuse among undocumented migrants, which could indirectly facilitate health (Wolff 2008).
	**Network extent and length of stay**	(28,30,31,51)	The longer history in the U.S. immigration of Mexicans is mentioned as potential facilitators at a national level (Held 2018).
	**Social intervention**	(6,7,10,11,13,20,21,28,30,37,49	Fear reduction through support from community-based health providers (Lane 2019).
			Involvement of NGOs, religious communities, and cultural doulas (Barkensjö 2018).
			Stable housing and cultural sensitivity training for healthcare providers (Liu 2019).

From our analysis, we find that whenever specific legal actions are promoted, like the protection of maternity, state reimbursement for healthcare providers, healthcare costs coverage for women and the enforcement of health rights, improved maternal and infant outcomes are presented as well. These include adequate prenatal care and more prenatal care visits,^42,51,69^ higher fetal weight gain,^42^ reduced inadequate care and increased diagnosis of gestational diabetes and pre-existing diabetes mellitus.^71^ With improved legal conditions, a lower frequency of adverse maternal conditions such as diabetes, gestational diabetes, higher BMI,^72^ is observed, as are increased rates of screenings and vaccines.^51^

Socio-cultural facilitators of access to healthcare include, among others, the presence of specific clinics dedicated to undocumented migrants or pregnant undocumented women. These clinics play a crucial role in safeguarding individuals’ identities, ensuring continuity of care, and concurrently enhance access to prenatal healthcare services. They also serve as potential entry points into the wider healthcare system, facilitating subsequent engagements with healthcare services.^72,73^ Similarly, any form of language support, including translators, interpreters or cultural mediators, optimises communication and, when present, data show improved attendance and compliance.^61^ As opposed to social interventions put in place to improve the experience and indirectly the health outcomes of undocumented migrants, personal characteristics also seem to play an important role in accessing healthcare in the first 1000 days. Healthier habits than those of the local population, or what could be called a cultural diversity in health literacy, allow a disadvantaged population to prevent health issues with or without healthcare access. In fact, the migrant population shows healthier patterns in alcohol consumption and healthier eating habits, helping women maintain good health during pregnancy and beyond.^35^

Furthermore, in the included studies we find that women that attend specific health clinics that provide access to maternal and infant healthcare in the first 1000 days also have fewer C-sections compared to the local population and no significant differences are found with respect to birth weight, delivery at term, instrumented delivery, and Apgar score.^73^ Similarly, as shown in a north American study, having a prior cultural understanding of the health interventions during the first 1000 days of life and healthcare systems of the host country allows undocumented migrants to easily adapt to and have no measurable differences in low birth weight between US born, foreign-born documented, and undocumented women.^66^ In five included articles, no facilitators are discussed.^27,54,63,74,75^

## Discussion

Even though international organisations such as the WHO, UN, and WMA consider the provision of equitable and accessible healthcare for everyone to be a human right,^1–3^ undocumented migrants face a multitude of barriers when accessing maternal and infant healthcare in the first 1000 days of life. This systematic review demonstrates that there are a number of complex challenges that significantly influence the healthcare experiences and outcomes of this vulnerable population. Against the many barriers, several facilitators have been identified that offer potential solutions to these obstacles and envision the right to healthcare for all.

While our review focuses specifically on undocumented migrant women and their infants during the first 1000 days of life, many of the barriers identified overlap with those reported in the broader literature on undocumented migrants in general. Another systematic review of the literature in fact has similarly pointed out that undocumented migrants generally refrain from accessing healthcare due to lack of financial resources and health insurance, fear, language and cultural discrepancies, discrimination, bureaucratic requirements, anti-immigrant rhetoric, and policy-related barriers.^76^ When then focusing specifically on women, we have found an interesting example from Sweden, conceptualising how adverse obstetric outcomes among immigrant women in high-income settings are directly impacted by three layers of delay in healthcare access. These encompass (1) delay in the decision to seek care, (2) delay in reaching care, and (3) delay in receiving adequate care.^77^ Our findings align with this framework, showing that legal, financial, cultural, and systemic obstacles often compound across all three stages. However, our targeted focus on the first 1000 days allows us to highlight how these delays not only affect obstetric outcomes but also shape early child development, maternal mental health, and long-term well-being. By applying a lifecycle perspective, our analysis adds depth to existing frameworks and emphasises the intergenerational impact of inadequate maternal and infant healthcare access.

Given the relevance of the results of our systematic review and to better contextualise our results, in the next paragraphs we discuss the study results within Dahlgren and Whitehead's model of the social determinants of health.^78^ Dahlgren and Whitehead's model is a socioecological framework that organises health determinants and possible policy actions into five hierarchical levels. These levels cover the (i) socioeconomic, cultural, and environmental conditions, (ii) living and occupational circumstances, (iii) social and community connections, (iv) individual lifestyle aspects, and (v) age, gender, and inherent characteristics, the latter representing unchanging individual traits.^78^

At the first level of the “socioeconomic, cultural and environmental conditions”, we note that whenever legal actions are put in place to prevent timely and regular access to healthcare for undocumented migrants, the results are increased rates of complications during pregnancy, consequent adverse neonatal outcomes, and maternal behavioural stress responses.^79,80^ Poor healthcare access for mothers and their infants can be prevented by putting in place policies enforcing and protecting health rights of the migrant population. The human right to health, or the access to healthcare, should be independent of the legal status as noted by international organisations such as the WHO and the WMA.^1,2,19^ Supporting rather than criminalising has demonstrated clear health benefits for the state governments and populations in similar-yet different-communities like sex workers, drug users or homeless people.^81^ A number of private or public initiatives around the world ensure the protection of undocumented migrants seeking care and, over time, have demonstrated their effectiveness in caring for people in need and protecting their long-term health.^82,83^ Based on these experiences, new policies should be implemented, aimed at improving the general socioeconomic, cultural and environmental conditions to respect and ensure health rights.

Second, drawing from this, legally enabling the development of dedicated facilities improves healthcare services for underserved populations and ensures dignity at the “living and working conditions” level.^84^ Such dedicated clinics ensure continuity of care, mutual respect, and trust by overcoming the complexities of disabling administrative procedures to access health rights but also in minimising the fear and psychological stress connected with the irregular legal situation. Furthermore, maternal care often represents the best opportunity for familiarising with the healthcare system as well as the culture surrounding health, ensuring cultural integration, health literacy, and social care.^85^

Third, regular employment and dignified housing influence the “social and community networks” level that is documented to be well developed in migrant populations, specifically for its scope and survival. Being uprooted, migrants are more likely to look for networking opportunities in the hosting community.^86^ What is more, the extent of one’s networks brings, beside working opportunities, social support to navigate the hosting society in all its cultural do’s and don’ts and improves mental health.^86,87^

Fourth, the societal, legal, and community integration efforts are reflected at the “individual level” where, depending on the enabling or disabling context the undocumented person finds themself in, lifestyle habits are seen as barriers or facilitators. This is the case for nutritional patterns where adhering to traditional healthy eating could prevent the development of adverse conditions during pregnancy.^37,42,88^ Additionally, it extends to other cultural and educational components related to reproductive health. As reported in 2016 by Gottlieb and De Loache in their book “A World of Babies”, cultural approaches to pregnancy and childbirth among migrant mothers worldwide inspire women with a sense of resilience, strength, and mental well-being, thereby contributing to their overall long-term health.^89^

Finally, at the level of the unchanging “individual traits” like age or gender, when integrated into social and healthcare dedicated initiatives, these could be identified as resources rather than weaknesses. As a matter of fact, younger (yet not teenage) age is mentioned as a potential facilitator as it naturally minimises the risks of adverse outcomes connected to advanced parental age.^58,90^

To sum up, efforts primarily ensuring the inclusion of migrant populations in new public health policies at government level should be paired with local ones, mainly connected with tackling the cultural and rooted stigma surrounding undocumented migration. These patterns reflect the intricate and ethical relationship between access barriers, healthcare experiences, and specific maternal and fetal outcomes for undocumented migrants, emphasising the need for a holistic and respectful approach to address healthcare disparities in this population and promote a shift from exclusion and blame toward inclusion, support and visibility in host societies. While not precisely fitting the social determinants of health framework, and for this reason, not expressly outlined when discussing the results against this framework; we believe that ethical considerations of access to healthcare for vulnerable populations affect all these levels transversely.

Despite the many advantages in terms of knowledge and understanding of dynamics within a population which has historically received little attention, we acknowledge that our work has some limitations. First and foremost, the geographic focus we decided to concentrate on included only literature from OECD host countries. This decision was mainly taken to allow comparison of results among similar healthcare systems in medium- to high-income countries, yet the dominance of US-based studies may affect the generalisability of the findings. Additionally, we acknowledge the constant difficulty in identifying undocumented migrants in data analysis, as authors and authorities rarely share a comprehensive definition that enables a straightforward data analysis. Another important limitation is that none of the included studies explicitly employed the first 1000 days of life framework, making narrative synthesis a picture of a scattered literature. Finally, we do not presume our review to be comprehensive as studies not indexed in the databases selected as well as those in grey literature have not received our attention.

## Conclusion

This is to our knowledge the first systematic review emphasising the barriers and facilitators in place for accessing maternal and infant healthcare in the first 1000 days, or from conception until the end of the child’s second year among undocumented migrant populations. Current literature primarily focuses on maternal well-being, while infant outcomes receive comparatively little attention. For this reason, future research should prioritise its focus on the long-term health outcomes of infants born to undocumented migrant mothers. More studies are also needed on maternal postnatal healthcare access and, in particular, how healthcare experiences differ based on geographic locations and implementation of identified facilitators. A comparison of such findings to evaluate the effectiveness of given interventions for undocumented mothers and infants using the first 1000 days framework must also be investigated.

Policy makers must consider the consequences of preventing timely and adequate healthcare access to undocumented mothers and their infants. Successful inclusive and human rights-based models in various countries have demonstrated measurable benefits for both migrants and host communities. Literature to date suggests that governments and public health institutions implement policies that: (1) grant regular and unconditional prenatal and postnatal healthcare access regardless of legal status; (2) invest in culturally adapted perinatal services and community-based outreach; (3) remove administrative complexities to access care; and (4) legally protect undocumented women from criminalisation when seeking healthcare.

As we advance towards the WHO’s universal health coverage and the UN’s 2030 agenda for sustainable development, with its 17 Sustainable Development Goals and 169 associated targets, the right to healthcare must be respected. This entails implementing healthcare access strategies to foster the well-being of pregnant migrant women and their children without discrimination based on legal status, origin or social circumstances.

## Author contributions

Conceptualisation: CM, NBN, TW. Methodology: CM, NBN, TW. Investigation: CM, NBN, TW. Data Curation: CM, NBN. Formal Analysis/Interpretation: CM, NBN. Validation: BSE, QC, TW. Writing – Original Draft: CM. Writing – Review & Editing: NBN, TW, BSE. All authors critically revised the manuscript for important intellectual content and approved the final version for publication.

## Supplementary Material

Supplemental material: Full search strategies for all consulted databases.
