# Supplementary material for: Facilitators and barriers of maternal and infant healthcare access for undocumented migrants in the first 1000 days of life: a systematic review of the literature
Source: Sex Reprod Health Matters. 2025 Sep 17;33(1):2560189. doi: 10.1080/26410397.2025.2560189 (PMC12538716; doi:10.1080/26410397.2025.2560189)
Supplement: Supplemental material: Full search strategies for all consulted databases. [file ZRHM_A_2560189_SM6569.docx]

**Supplementary file 1: Full search strategies for all consulted databases**

**Table 1. Full search strategy for Embase**

| **Context**  **AND**  **Outcome** | ('undocumented immigrant'/exp OR 'illegal migrants'/exp OR 'illegal migration'/exp OR 'clandestine'/exp OR 'undocumented immigrant*':ab,ti OR 'undocumented migrant*':ab,ti OR 'unregistered person*':ab,ti OR 'unregistered immigrant*':ab,ti OR 'unregistered migrant*':ab,ti OR 'illegal immigrant*':ab,ti OR 'illegal migrant*':ab,ti OR 'irregular immigrant*':ab,ti OR 'irregular migrant*':ab,ti OR 'unauthorised immigrant*':ab,ti OR 'unauthorised migrant*':ab,ti OR 'unauthorised person*':ab,ti OR 'unauthorized immigrant*':ab,ti OR 'unauthorized migrant*':ab,ti OR 'unauthorized person*':ab,ti OR 'undocumented wom*':ab,ti OR 'illegal wom*':ab,ti OR 'irregular wom*':ab,ti OR 'undocumented child*':ab,ti OR 'sans papier':ab,ti OR 'clandestin*':ab,ti OR 'lack of legal status':ab,ti OR 'paperless':ab,ti)  AND  ('maternal care'/exp OR 'prenatal care'/exp OR 'puerperium'/exp OR 'postnatal care'/exp OR 'perinatal care'/exp OR 'postpartum'/exp OR 'pregnancy'/exp OR 'reproductive health'/exp OR 'child health care'/exp OR 'reproductive health women'/exp OR 'maternal health service'/exp OR 'maternal care':ab,ti OR 'maternal healthcare':ab,ti OR 'prenatal care':ab,ti OR 'prenatal healthcare':ab,ti OR 'antenatal care':ab,ti OR 'antenatal healthcare':ab,ti OR 'postpartum care':ab,ti OR 'postpartum healthcare':ab,ti OR 'postnatal care':ab,ti OR 'postnatal healthcare':ab,ti OR 'perinatal care':ab,ti OR 'perinatal healthcare':ab,ti OR 'perinatal health':ab,ti OR 'child healthcare':ab,ti OR 'child health care':ab,ti OR 'infant healthcare':ab,ti OR 'pregnancy':ab,ti OR 'pregnancy care':ab,ti OR 'pregnancy healthcare':ab,ti OR 'maternity service*':ab,ti OR 'reproductive health':ab,ti OR 'reproductive health service*':ab,ti OR 'maternal health service*':ab,ti OR 'puerperium':ab,ti OR 'pregnancy health':ab,ti OR 'maternity care':ab,ti) |
| --- | --- |
| **With the applied filters: “time” (** **01.01.2000-31.10.2024), “human”, “article type” (Article, article in press, data papers, pre-print, review, short survey), and “language” (English, French, Spanish, Italian and German).** | |

**Table 2. Full search strategy for Pubmed incl. Medline**

| **Context**  **AND**  **Outcome** | ((("Undocumented Immigrants"[Mesh] OR "undocumented immigrant*"[Title/Abstract] OR "undocumented migrant*"[Title/Abstract] OR "unregistered person*"[Title/Abstract] OR "unregistered immigrant*"[Title/Abstract] OR "unregistered migrant*"[Title/Abstract] OR "illegal immigrant*"[Title/Abstract] OR "illegal migrant*"[Title/Abstract] OR "irregular immigrant*"[Title/Abstract] OR "irregular migrant*"[Title/Abstract] OR "unauthorised immigrant*"[Title/Abstract] OR "unauthorised migrant*"[Title/Abstract] OR "unauthorised person*"[Title/Abstract] OR "unauthorized immigrant*"[Title/Abstract] OR "unauthorized migrant*"[Title/Abstract] OR "unauthorized person*"[Title/Abstract] OR "undocumented wom*"[Title/Abstract] OR "illegal wom*"[Title/Abstract] OR "irregular wom*"[Title/Abstract] OR "undocumented child*"[Title/Abstract] OR "sans papier"[Title/Abstract] OR "clandestin*"[Title/Abstract] OR "lack of legal status"[Title/Abstract] OR "paperless"[Title/Abstract]))  AND  (("Maternal Health"[Mesh] OR "Maternal Health Services"[Mesh] OR "Maternal-Child Health Centers"[Mesh] OR "Child Health"[Mesh] OR "Infant Care"[Mesh] OR "Child Health Services"[Mesh] OR "Maternal-Child Health Services"[Mesh] OR "Prenatal Care"[Mesh] OR "Postnatal Care"[Mesh] OR "Perinatal Care"[Mesh] OR "Postpartum Period"[Mesh] OR "Pregnancy"[Mesh] OR "Reproductive Health"[Mesh] OR "Reproductive Health Services"[Mesh] OR "Obstetrics"[Mesh] OR "maternal care"[Title/Abstract] OR "maternal healthcare"[Title/Abstract] OR "prenatal care"[Title/Abstract] OR "prenatal healthcare"[Title/Abstract] OR "antenatal care"[Title/Abstract] OR "antenatal healthcare"[Title/Abstract] OR "postpartum care"[Title/Abstract] OR "postpartum healthcare"[Title/Abstract] OR "postnatal care"[Title/Abstract] OR "postnatal healthcare"[Title/Abstract] OR "perinatal care"[Title/Abstract] OR "perinatal healthcare"[Title/Abstract] OR "perinatal health"[Title/Abstract] OR "child healthcare"[Title/Abstract] OR "child health care"[Title/Abstract] OR "infant healthcare"[Title/Abstract] OR "pregnancy"[Title/Abstract] OR "pregnancy care"[Title/Abstract] OR "pregnancy healthcare"[Title/Abstract] OR "maternity service*"[Title/Abstract] OR "reproductive health"[Title/Abstract] OR "reproductive health service*"[Title/Abstract] OR "maternal health service*"[Title/Abstract] OR "puerperium"[Title/Abstract] OR "pregnancy health"[Title/Abstract] OR "maternity care"[Title/Abstract]))) |
| --- | --- |
| **With the applied filters: “time” (** **01.01.2000-31.10.2024), “human”, and “language” (English, French, Spanish, Italian and German).** | |

**Table 3. Full search strategy for Scopus**

| **Context**  **AND**  **Outcome** | ( TITLE-ABS-KEY ( "undocumented immigrant*" OR "undocumented migrant*" OR "unregistered person*" OR "unregistered immigrant*" OR "unregistered migrant*" OR "illegal immigrant*" OR "illegal migrant*" OR "irregular immigrant*" OR "irregular migrant*" OR "unauthorised immigrant*" OR "unauthorised migrant*" OR "unauthorised person*" OR "unauthorized immigrant*" OR "unauthorized migrant*" OR "unauthorized person*" OR "undocumented wom*" OR "illegal wom*" OR "irregular wom*" OR "undocumented child*" OR "sans papier" OR "clandestin*" OR "lack of legal status" OR "paperless" )  AND  TITLE-ABS-KEY ( "maternal care" OR "maternal healthcare" OR "prenatal care" OR "prenatal healthcare" OR "antenatal care" OR "antenatal healthcare" OR "postpartum care" OR "postpartum healthcare" OR "postnatal care" OR "postnatal healthcare" OR "perinatal care" OR "perinatal healthcare" OR "perinatal health" OR "child healthcare" OR "child health care" OR "infant healthcare" OR "pregnancy" OR "pregnancy care" OR "pregnancy healthcare" OR "maternity service*" OR "reproductive health" OR "reproductive health service*" OR "maternal health service*" OR "puerperium" OR "pregnancy health" OR "maternity care" ) ) |
| --- | --- |
| **With the applied filters: “time” (** **01.01.2000-31.10.2024) and “language” (English, French, Spanish, Italian and German).** | |

**Table 4. Full search strategy for PsycInfo**

| **Context**  **AND**  **Outcome** | (('undocumented immigrant*' or 'undocumented migrant*' or 'unregistered person*' or 'unregistered immigrant*' or 'unregistered migrant*' or 'illegal immigrant*' or 'illegal migrant*' or 'irregular immigrant*' or 'irregular migrant*' or 'unauthorised immigrant*' or 'unauthorised migrant*' or 'unauthorised person*' or 'unauthorized immigrant*' or 'unauthorized migrant*' or 'unauthorized person*' or 'undocumented wom*' or 'illegal wom*' or 'irregular wom*' or 'undocumented child*' or 'sans papier' or 'clandestin*' or 'lack of legal status' or 'paperless').ti. or ('undocumented immigrant*' or 'undocumented migrant*' or 'unregistered person*' or 'unregistered immigrant*' or 'unregistered migrant*' or 'illegal immigrant*' or 'illegal migrant*' or 'irregular immigrant*' or 'irregular migrant*' or 'unauthorised immigrant*' or 'unauthorised migrant*' or 'unauthorised person*' or 'unauthorized immigrant*' or 'unauthorized migrant*' or 'unauthorized person*' or 'undocumented wom*' or 'illegal wom*' or 'irregular wom*' or 'undocumented child*' or 'sans papier' or 'clandestin*' or 'lack of legal status' or 'paperless').ab.)  AND  (exp Prenatal Care/ or exp Postnatal Period/ or exp Antepartum Period/ or exp Perinatal Period/ or exp Pregnancy/ or exp Child Care/ or exp Obstetrics/) OR (('maternal care' or 'maternal healthcare' or 'prenatal care' or 'prenatal healthcare' or 'antenatal care' or 'antenatal healthcare' or 'postpartum care' or 'postpartum healthcare' or 'postnatal care' or 'postnatal healthcare' or 'perinatal care' or 'perinatal healthcare' or 'perinatal health' or 'child healthcare' or 'child health care' or 'infant healthcare' or 'pregnancy' or 'pregnancy care' or 'pregnancy healthcare' or 'maternity service*' or 'reproductive health' or 'reproductive health service*' or 'maternal health service*' or 'puerperium' or 'pregnancy health' or 'maternity care').ti.) or (('maternal care' or 'maternal healthcare' or 'prenatal care' or 'prenatal healthcare' or 'antenatal care' or 'antenatal healthcare' or 'postpartum care' or 'postpartum healthcare' or 'postnatal care' or 'postnatal healthcare' or 'perinatal care' or 'perinatal healthcare' or 'perinatal health' or 'child healthcare' or 'child health care' or 'infant healthcare' or 'pregnancy' or 'pregnancy care' or 'pregnancy healthcare' or 'maternity service*' or 'reproductive health' or 'reproductive health service*' or 'maternal health service*' or 'puerperium' or 'pregnancy health' or 'maternity care').ab.) |
| --- | --- |
| **With the applied filters: “time” (01.01.2000-31.10.2024) and “language” (English, French, Spanish, Italian and German).** | |

**Table 5. Full search strategy for CINAHL**

| **Context**  **AND**  **Outcome** | ( TI ( 'undocumented immigrant*' OR 'undocumented migrant*' OR 'unregistered person*' OR 'unregistered immigrant*' OR 'unregistered migrant*' OR 'illegal immigrant*' OR 'illegal migrant*' OR 'irregular immigrant*' OR 'irregular migrant*' OR 'unauthorised immigrant*' OR 'unauthorised migrant*' OR 'unauthorised person*' OR 'unauthorized immigrant*' OR 'unauthorized migrant*' OR 'unauthorized person*' OR 'undocumented wom*' OR 'illegal wom*' OR 'irregular wom*' OR 'undocumented child*' OR 'sans papier' OR 'clandestin*' OR 'lack of legal status' OR 'paperless' ) OR AB ( 'undocumented immigrant*' OR 'undocumented migrant*' OR 'unregistered person*' OR 'unregistered immigrant*' OR 'unregistered migrant*' OR 'illegal immigrant*' OR 'illegal migrant*' OR 'irregular immigrant*' OR 'irregular migrant*' OR 'unauthorised immigrant*' OR 'unauthorised migrant*' OR 'unauthorised person*' OR 'unauthorized immigrant*' OR 'unauthorized migrant*' OR 'unauthorized person*' OR 'undocumented wom*' OR 'illegal wom*' OR 'irregular wom*' OR 'undocumented child*' OR 'sans papier' OR 'clandestin*' OR 'lack of legal status' OR 'paperless' ) )  AND  ( (MH "Maternal-Child Care+") OR (MM "Prenatal Care") OR (MH "Postnatal Care+") OR (MH "Postnatal Period+") OR (MH "Child Health Services+") OR (MM "Child Health") OR (MM "Maternal-Child Health") OR (MH "Pregnancy+") OR (MH "Maternal Health Services+") OR (MM "Reproductive Health") OR (MM "Puerperium") OR (TI ( 'maternal care' OR 'maternal healthcare' OR 'prenatal care' OR 'prenatal healthcare' OR 'antenatal care' OR 'antenatal healthcare' OR 'postpartum care' OR 'postpartum healthcare' OR 'postnatal care' OR 'postnatal healthcare' OR 'perinatal care' OR 'perinatal healthcare' OR 'perinatal health' OR 'child healthcare' OR 'child health care' OR 'infant healthcare' OR 'pregnancy' OR 'pregnancy care' OR 'pregnancy healthcare' OR 'maternity service*' OR 'reproductive health' OR 'reproductive health service*' OR 'maternal health service*' OR 'puerperium' OR 'pregnancy health' OR 'maternity care' ) OR (AB ( 'maternal care' OR 'maternal healthcare' OR 'prenatal care' OR 'prenatal healthcare' OR 'antenatal care' OR 'antenatal healthcare' OR 'postpartum care' OR 'postpartum healthcare' OR 'postnatal care' OR 'postnatal healthcare' OR 'perinatal care' OR 'perinatal healthcare' OR 'perinatal health' OR 'child healthcare' OR 'child health care' OR 'infant healthcare' OR 'pregnancy' OR 'pregnancy care' OR 'pregnancy healthcare' OR 'maternity service*' OR 'reproductive health' OR 'reproductive health service*' OR 'maternal health service*' OR 'puerperium' OR 'pregnancy health' OR 'maternity care' )) ) |
| --- | --- |
| **With the applied filters: “time” (01.01.2000-31.10.2024), “article type” (academic journals), and “language” (English, French, Spanish, Italian and German).** | |
